# Falls predict faster progression to Alzheimer's dementia

**DOI:** 10.1002/alz.71177

**Published:** 2026-02-07

**Authors:** Audrey A. Keleman, Melody Li, Julie K. Wisch, Rebecca M. Bollinger, Melissa J. Krauss, Elizabeth A. Grant, Tammie L. S. Benzinger, Beau M. Ances, John C. Morris, Susan L. Stark

**Affiliations:** ^1^ Program in Occupational Therapy Washington University School of Medicine St. Louis Missouri USA; ^2^ Department of Veterans Affairs Eastern Colorado Geriatric Research, Education, and Clinical Center Aurora Colorado USA; ^3^ Department of Physical Medicine and Rehabilitation University of Colorado School of Medicine Aurora Colorado USA; ^4^ Department of Neurology Washington University School of Medicine St. Louis Missouri USA; ^5^ Institute for Informatics Data Science and Biostatistics (I2DB) Washington University School of Medicine St. Louis Missouri USA; ^6^ Department of Radiology Washington University School of Medicine St. Louis Missouri USA

**Keywords:** aging, Alzheimer disease, amyloid, dementia, falls

## Abstract

**INTRODUCTION:**

In preclinical Alzheimer's disease (AD), amyloid accumulates in the brain while individuals remain cognitively unimpaired (Clinical Dementia Rating^®^ [CDR] = 0). Differentiating trajectories of healthy aging and preclinical AD is challenging as both are associated with age‐related impairments (e.g., falls).

**METHODS:**

Longitudinal cohort study. We monitored falls for 1 year among 125 CDR 0 older adults and assessed preclinical AD status (amyloid). We continued to evaluate CDR annually (median 10 years). The cohort was grouped: Preclinical AD−Fall−, Preclinical AD−Fall+, Preclinical AD+Fall−, and Preclinical AD+Fall+. Survival analysis examined time to progression to CDR 1 (mild dementia) by group.

**RESULTS:**

Participants were 74 years (mean) at baseline, 62% female, 96% White. Preclinical AD+Fall+ progressed to CDR 1 most rapidly. Preclinical AD−Fall− progressed least quickly. Preclinical AD+Fall− and Preclinical AD−Fall+ had similar progression rates.

**DISCUSSION:**

Falls may associate with faster progression of AD dementia, potentially reflecting motor and gait dysfunction intrinsic to disease progression.

## Highlights

- Falls predicted faster progression to dementia symptoms.
- Those without falls or amyloid burden progressed to dementia least quickly.
- Falls may serve as early markers of motor dysfunction in Alzheimer's disease.

## 1 BACKGROUND

Alzheimer's disease (AD) is a progressive neurodegenerative disorder with profound consequences for affected individuals, families, and healthcare systems.^1^ AD begins with a decades‐long preclinical stage that is characterized by amyloid accumulation in the brain while individuals remain cognitively unimpaired (Clinical Dementia Rating^®^ [CDR] = 0).^2^, ^3^ Although cognitive symptoms are not yet apparent, mounting evidence suggests that sensory and motor dysfunction precede cognitive changes,^4^, ^5^, ^6^ making these domains promising candidates for early disease markers.

Falls represent a significant health concern among older adults, with more than one‐third of those aged 65 years or older experiencing a fall annually.^7^, ^8^, ^9^ Falls are a leading cause of injury, injury‐related mortality, long‐term disability, and premature placement into skilled nursing facilities among older adults.^8^ While falls are commonly attributed to age‐related factors such as balance and gait impairments, sarcopenia, vision impairment, and polypharmacy, their potential connection to neurodegenerative processes is understudied.^7^, ^9^, ^10^, ^11^, ^12^, ^13^ Growing evidence indicates that individuals with AD pathology, even in the absence of cognitive symptoms, may have an increased fall risk due to early motor system impairments.^4^, ^14^, ^15^, ^16^, ^17^, ^18^, ^19^ These impairments, coupled with subtle cognitive changes, may contribute to a greater vulnerability to fall‐related injuries and may serve as a clinical marker for impending progression to dementia.^20^ Monitoring individuals with preclinical AD pathology more closely for falls may be indicated, as consequences of falls are devastating, and screening for falls and fall risk can be done relatively easily by patients and care partners. On the other hand, falls may serve as a pathway to identify individuals for whom more costly tests may be indicated (e.g., blood tests, imaging for AD pathology).

The relationship between preclinical AD and falls is complex, given that aging affects both cognitive and motor function.^6^, ^21^, ^22^, ^23^ Disentangling changes related to AD pathology from those associated with healthy aging is essential for developing useful disease‐specific biomarkers. Motor impairments are common in both normal aging and preclinical AD,^21^, ^22^ but the rate and pattern of decline may differ between the two processes. Studying falls as a clinical marker of AD may enhance understanding and predictions of progression of the disease by capturing a wider array of dysfunction in the early period, while cognition is not yet impaired. Falls are real‐world events that could prompt referral and intervention when other early AD markers are often not initiated until later in the disease process when cognitive impairments are observed.^24^

This study investigates whether falls are associated with an increased risk of progression to mild dementia (CDR 1) in older adults with and without preclinical AD pathology. In a longitudinal cohort of cognitively unimpaired older adults (all CDR 0 at baseline), we assessed amyloid status through positron emission tomography (PET) imaging once at baseline, tracked falls prospectively over 1 year (starting at baseline for 12 months), and continued to evaluate CDR annually. We hypothesized that individuals with both amyloid burden and a history of falls (Preclinical AD+Fall+) would experience the fastest progression to dementia, followed by those with either amyloid burden or falls alone (Preclinical AD+Fall−, Preclinical AD−Fall+).

**RESEARCH IN CONTEXT**

1. **Systematic review**: We used databases such as PubMed to review literature and place findings in context. While there are studies that demonstrate motor, balance, and falls in preclinical Alzheimer's disease (AD), to our knowledge, none have examined time to dementia symptom onset using preclinical AD status and falls.
2. **Interpretation**: Our findings indicate that falls are associated with faster progression to mild dementia in older adults. Individuals with both amyloid burden and a history of falls showed the most rapid progression to mild dementia. This suggests falls may serve as an early clinical marker of AD progression and may reflect underlying motor and gait dysfunction intrinsic to the disease.
3. **Future directions**: Future research should focus on elucidating the mechanistic links between falls, motor dysfunction, and amyloid accumulation in preclinical AD, and their relationship with progression to dementia symptoms. It should also be explored whether fall prevention interventions can delay the onset of dementia in at‐risk individuals.

## 2 METHODS

### 2.1 Participants

Community‐dwelling individuals enrolled in longitudinal studies of memory and aging at the Knight Alzheimer Disease Research Center (ADRC) were invited to enroll in this study if they: (1) were age 65 years or older, (2) were cognitively unimpaired evidenced by a CDR^25^ of 0 at their most recent annual assessment (within 1 year of enrollment in this study), and (3) underwent an amyloid PET imaging study within 2 years of enrollment in this study. Further details of this study have been published previously.^14^

The Institutional Review Board at Washington University in St. Louis approved this study. The study was performed in accordance with the ethical standards as laid down in the 1964 Declaration of Helsinki and its later amendments.

### 2.2 Study design

#### 2.2.1 First year of study

Recruitment, assessment of baseline participant characteristics and fall covariates, and 12 months of fall data collection took place from May 2010 to November 2011. Self‐reported demographic information was collected in accordance with the Uniform Data Set.^26^ Cognition was assessed using the Mini Mental State Examination (MMSE), an 11‐item evaluation of cognitive impairment across five domains: orientation, registration, attention and calculation, recall, and language.^27^

#### 2.2.2 Longitudinal follow‐up

After 12 months of fall monitoring, participants continued annual clinical assessment at the Knight ADRC for up to 12 years. This annual clinical assessment included CDR ascertainment and attrition data such as death or withdrawal from the study.

### 2.3 Measures

#### 2.3.1 CDR (annual clinical assessment)

CDR was the primary outcome in this study. During the annual clinical assessment, trained clinicians obtained a CDR^25^ following a standardized protocol in an interview with the participant and a collateral source.^28^ The CDR is based on performance across six domains—memory, orientation, judgment and problem solving, community affairs, home and hobbies, and personal care—and is known for its established reliability.^25^ CDR scores range from 0 (cognitively unimpaired) to 3 (severe dementia).^25^ CDR 1 represents mild dementia symptoms.

#### 2.3.2 Prospective fall ascertainment (12 months at baseline)

Each participant received a 12‐month calendar journal to record whether a fall occurred each day. The journals were personalized, incorporating birthdays and other important personal dates to help participants recall when a fall occurred.^29^ The calendar journals also included space for participants to provide details of any falls that took place (e.g., time of day, what they were doing at the time, direction of the fall, what happened after the fall). Participants received training via telephone on how to use the journal. A fall was defined as any unintentional descent to the floor, ground, or an object below knee level.^30^ Participants returned their calendar pages via mail on a monthly basis, and fall records were reviewed.^30^ If a fall was reported, a member of the research team interviewed the participant via telephone to confirm that it aligned with the operational definition of a fall. Participants received a gift card after returning a completed calendar journal page each month. Fall severity details were included in fall reporting and interview data. A published algorithm for fall severity was used to classify participants upon completion of 1 year of fall monitoring: (0) no falls, (1) one fall without serious injury that did not require medical attention, (2) more than one fall or a fall with minor injury that required medical attention but did not result in hospital admission, (3) a fall with a major injury that led to hospital admission.^8^ This method of prospective fall ascertainment affords strategies to report a fall and important details close to its occurrence (calendar journal) and a maximum 1‐month recall period (active asking about falls).^29^ These are both strategies that have been associated with greater validity in fall monitoring among community‐dwelling older adults.^31^, ^32^, ^33^

#### 2.3.3 Amyloid positron emission tomography (baseline)

Positron emission tomography (PET) imaging was performed within 2 years of enrollment in this study, following previously described methods.^34^, ^35^ Participants were injected with 12–15 mCi of [11C] Pittsburgh compound B, and dynamic scans were collected. The time window for post‐injection quantification was 30–60 minutes. A PET Unified Pipeline (github.com/ysu001/PUP) was used for data processing. In short, a region of interest (ROI) segmentation approach was applied using FreeSurfer 5.3 (Martinos Center for Biomedical Imaging, Charlestown, Massachusetts, USA). A tissue mask was generated based on ROI segmentation.^34^ The standard uptake ratio was calculated for each ROI, using the cerebellar gray matter as a reference region. A summary measure, the mean cortical standardized uptake value ratio (SUVR), was derived from cortical regions affected by AD after partial‐volume correction. Participants were identified as having preclinical AD if they had an SUVR greater than 1.42 (16.4 Centiloids).^34^, ^36^

#### 2.3.4 Fall risk factors (baseline)

Each participant completed a 10–15‐min telephone interview to assess factors previously identified as being related to falls and to characterize the sample. Problematic alcohol behavior was evaluated using the Short Michigan Alcoholism Screening Test–Geriatric Version (SMAST‐G), a 10‐item interview validated for older adults.^37^ A score of 2 or higher indicates likely problematic alcohol behaviors. Limitations in the ability to perform activities of daily living (ADL) and instrumental ADL were measured using the Older American Resources and Services (OARS) ADL scale.^38^ This scale asks respondents about their ability to perform 14 activities, with responses scored on a 0–2 scale. Higher scores indicate greater independence. The number of prescription medications taken was also assessed. Participants reported their prescription medications and dosages, and the total number of medications taken monthly was calculated. An in‐person neurological examination evaluated gait, classifying it as either normal or abnormal.

### 2.4 Statistical analysis

We stratified participants by preclinical AD status and fall occurrence from the first year of the study, creating four groups for analyses: Preclinical AD−Fall−, Preclinical AD−Fall+, Preclinical AD+Fall−, and Preclinical AD+Fall+. Preclinical AD−Fall− served as the reference group. Descriptive statistics were calculated for baseline characteristics, fall data, and longitudinal outcomes (progression to CDR 1, attrition). Differences across groups were assessed using Kruskal–Wallis tests for continuous variables and Pearson's chi‐squared or Fisher's exact tests for categorical variables.

To examine time‐to‐event outcomes, we used Kaplan–Meier survival analysis to investigate the time from baseline (enrollment) to progression to CDR 1 (mild dementia) across groups. Comparisons between groups were made using a log‐rank test to provide an unadjusted comparison and facilitate visual interpretation of group differences. Pairwise comparisons of survival curves between groups were also performed using the log‐rank test and adjusted for multiple comparisons using the Benjamini–Hochberg method. We then applied Cox proportional hazards regression to estimate hazard ratios (HRs) while adjusting for age, allowing for a more comprehensive evaluation of associations and control for covariates.

The event date was defined as the first annual clinical assessment at which a participant received a CDR 1 rating (mild dementia symptoms). We used CDR 1 instead of CDR 0.5 because of the risk of CDR reversion.^39^ Participants who remained CDR < 1 throughout follow‐up and died or withdrew were censored at their last annual clinical assessment (the last time they were known to be CDR < 1). Those with changes in follow‐up protocol, most commonly due to virtual visits during the coronavirus disease 2019 (COVID‐19) pandemic in 2020, were censored at their last in‐person annual assessment to ensure consistency in the primary outcome.

In Cox models, ties were handled using the Efron method. The proportional hazards assumption was tested using Schoenfeld residuals and verified for all variables in both unadjusted and adjusted models (*p *> 0.05 for all covariates and the global test). Harrell's C‐index was used to assess model concordance. Final unadjusted and adjusted models are reported. Statistical tests were two‐tailed with an alpha threshold of 0.05, except for pairwise comparisons, which were adjusted using the Benjamini–Hochberg method. Sensitivity analyses are included in the Supplementary Material. Analyses were performed and figures were generated using R^40^ with psych,^41^ tidyverse,^42^ gtsummary,^43^ survival,^44^ and ggsurvplot.^45^

## 3 RESULTS

Participants (*N* = 125) were, on average, aged 74 years (standard deviation [SD] 6), mostly female (62%), White (96%), and highly educated (mean 15 years, SD 3; Table 1). Seventy‐four participants (59%) experienced at least one fall in the 1 year of fall monitoring. The median number of falls per person over the year was 1 (range 0–12; 33 [26%] had one fall, 22 [18%] had two falls, 13 [10%] had three falls, four [3%] had four falls, and one had six, seven, and 12 falls [< 1%, respectively]). Thirty‐seven participants (30%) had preclinical AD based on amyloid PET. The number of participants per group stratified by preclinical AD and fall status were as follows: Preclinical AD−Fall− (*n* = 35), Preclinical AD−Fall+ (*n* = 53), Preclinical AD+Fall− (*n* = 16), and Preclinical AD+Fall+ (*n* = 21; Table 1).

**TABLE 1:** Participant characteristics (grouped by faller and preclinical AD status).

Parameter	Total sample *N* = 125	Preclinical AD‐Fall− *n* = 35	Preclinical AD‐Fall+ *n* = 53	Preclinical AD+Fall− *n* = 16	Preclinical AD+ Fall+ *n* = 21	*p*‐Value^*^
**Baseline characteristics**						
Age, in years, mean (SD)	74 (6)	74 (6)	74 (5)	76 (8)	75 (5)	0.5
Female, *n* (%)	78 (62%)	20 (57%)	38 (72%)	8 (50%)	12 (57%)	0.3
Education, in years, mean (SD)	15 (3)	15 (3)	16 (3)	16 (2)	16 (3)	>0.9
**Race, *n* (%)**						0.2
Black	4 (3%)	1 (3%)	1 (2%)	2 (12%)	0 (0%)	
White	120 (96%)	33 (94%)	52 (98%)	14 (88%)	21 (100%)	
More than 1	1 (1%)	1 (3%)	0 (0%)	0 (0%)	0 (0%)	
MMSE, mean (SD)	29 (1)	29 (1)	29 (1)	29 (1)	29 (2)	0.1
*APOE*4 Carrier, *n* (%)	37 (30%)	9 (26%)	6 (11%)	8 (50%)	14 (67%)	<0.001
No. of medications, mean (SD)	4 (3)	3 (2)	4 (3)	3 (2)	3 (2)	0.8
ADL performance, mean (SD)	27.5 (0.8)	27.7 (0.9)	27.5 (0.8)	27.6 (1.0)	27.3 (0.7)	0.01
Gait abnormality, *n* (%)	4 (3%)	2 (6%)	1 (2%)	0 (0%)	1 (5%)	0.6
Problematic alcohol behavior, ^†^ *n* (%)	9 (7%)	3 (8.6%)	5 (9.4%)	0 (0%)	1 (4.8%)	0.8
Problematic alcohol behavior, ^†^ mean (SD)	0 (1)	0 (1)	0 (1)	0 (0)	0 (1)	0.1
**Falls (prospectively collected in first year of study)**						
Falls (total in one year), mean (SD)	1 (2)	N/A (0)	2 (1)	N/A (0)	3 (2)	0.03
Median (range)	1 (1–12)		1 (1–7)		2 (1–12)	
Fall Severity (highest in one year),^††^				0.01
*n* (%)				
One fall without injury	29 (23%)	25 (47%)	3 (14%)	
>1 fall or fall with injury	46 (37%)	28 (53%)	18 (86%)	
Longitudinal follow‐up (annual CDR and attrition)						
Progressed to CDR 1 during follow‐ up	25 (20%)	1 (3%)	8 (15%)	6 (38%)	10 (48%)	<0.001
Attrition: Died during follow‐up	32 (26%)	8 (23%)	15 (28%)	5 (31%)	4 (19%)	0.8
Attrition: Withdrew from study during follow‐up	18 (14%)	9 (26%)	6 (26%)	2 (13%)	1 (5%)	0.2

Abbreviations: AD, Alzheimer's disease; ADL, activities of daily living; APOE4, apolipoprotein E4; CDR, Clinical Dementia Rating; N/A, not applicable; SD, standard deviation.

* Kruskal–Wallis's rank sum test; Pearson's *χ* ^2^ test; Fisher's exact test.

^†^ Measured using the Short Michigan Alcoholism Screening Test—geriatric version (SMAST‐G).^37^ Reported as frequency of participants who meet cutoff of two indicating problematic alcohol behavior and raw score.

^††^ *n* = 50 (40%) of total sample had no falls and therefore a 0 on fall severity; this level is not included in comparison of groups, as it is used to define the groups.

The four groups did not differ by age, gender, education, race, Mini‐Mental State Examination (MMSE), number of medications, gait abnormality, or likely problematic alcohol behaviors (Table 1). Preclinical AD+ groups had a higher percentage of apolipoprotein E4 (*APOE*4) carriers compared to Preclinical AD‐ groups. ADL performance was slightly worse in all groups compared to Preclinical AD−Fall− and was poorest in the Preclinical AD+Fall+ group. Fall severity differed across the two groups of fallers; participants with preclinical AD who fell (Preclinical AD+Fall+) were more likely to experience multiple falls or falls with injury compared to fallers without preclinical AD (Preclinical AD−Fall+; Table 1). Specifically, 86% of Preclinical AD+Fall+ participants had more than one fall or a fall resulting in injury, compared to 53% in Preclinical AD−Fall+ (*p *= 0.01).

The follow‐up period with annual CDR assessment was a median of 10.4 years, or 125 months (range 1–145 months). Thirty‐two participants (26%) died during the follow‐up period. Number of deaths and study attrition (withdrawal) did not differ significantly across groups (Table 1).

**FIGURE 1:**
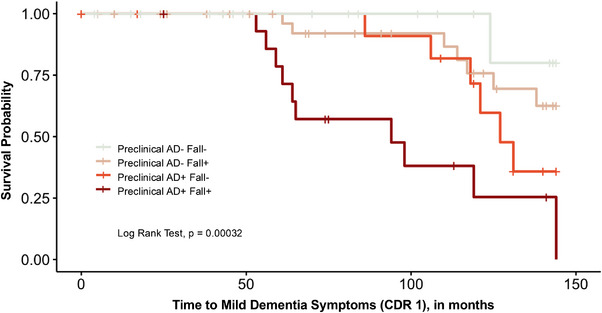
Time to clinical dementia rating 1 (mild dementia). Kaplan–Meier curves by group. Older adults with preclinical AD who fell (Preclinical AD+Fall+) progressed to mild dementia most quickly. Preclinical AD−Fall− progressed to mild dementia least quickly. Upon pairwise comparisons, a significant difference in survival was observed between the Preclinical AD−Fall− and Preclinical AD+Fall+ groups (adjusted *p* = 0.004) and between the Preclinical AD−Fall+ and Preclinical AD+Fall+ groups (adjusted *p* = 0.004). There were no other significant pairwise comparisons observed (Preclinical AD−Fall+ versus Preclinical AD−Fall− adjusted *p* = 0.319; Preclinical AD−Fall+ versus Preclinical AD+Fall− adjusted *p* = 0.311; Preclinical AD+Fall− versus Preclinical AD−Fall− adjusted *p* = 0.150; Preclinical AD+Fall+ versus Preclinical AD+Fall− adjusted *p* = 0.111). Preclinical AD+Fall− and Preclinical AD−Fall+ had similar progression rates. AD, Alzheimer's disease.

Progression to CDR 1 (mild dementia symptoms) was fastest in the Preclinical AD+Fall+ group, as demonstrated by the Kaplan‐Meier curves (Figure 1; log rank test *p *< 0.001). After accounting for censoring, 48% of Preclinical AD+Fall+ participants progressed to CDR 1, compared to 38% in Preclinical AD+Fall−, 15% in Preclinical AD−Fall+, and only 3% in the Preclinical AD−Fall− group. Upon pairwise comparisons, a significant difference in survival was observed between the Preclinical AD−Fall− and Preclinical AD+Fall+ groups (adjusted *p *= 0.004) and between the Preclinical AD−Fall+ and Preclinical AD+Fall+ groups (adjusted *p *= 0.004). There were no other significant pairwise comparisons observed (Preclinical AD−Fall+ versus Preclinical AD−Fall− adjusted *p *= 0.319; Preclinical AD−Fall+ versus Preclinical AD+Fall− adjusted *p *= 0.311; Preclinical AD+Fall− versus Preclinical AD−Fall− adjusted *p *= 0.150; Preclinical AD+Fall+ versus Preclinical AD+Fall− adjusted *p *= 0.111). Median survival times could not be estimated for some groups because more than 50% of participants remained CDR < 1 or were censored at the end of the follow‐up period. For Preclinical AD+Fall+, median survival was 94 months (mean 96 months); for Preclinical AD+Fall−, median survival was 127 months (mean 126 months); for Preclinical AD−Fall+, mean survival was 131 months; and for Preclinical AD−Fall−, mean survival was 140 months.

The Cox proportional hazards models (Table 2) revealed that, relative to Preclinical AD−Fall−, participants in the Preclinical AD+Fall+ group had the highest HR for progression to CDR 1 (HR = 21.3, 95% confidence interval [CI]: 2.7–166.9; *p *< 0.001), and this association remained after adjusting for age (HR = 26.9, 95% CI: 3.4–215.4; *p *< 0.001). Preclinical AD+Fall− participants also had a significantly higher risk of progression (HR = 15.1, 95% CI: 1.8–125.7; *p *= 0.01), which increased after adjusting for age (HR = 17.4, 95% CI: 2.1–146.8; *p *= 0.01). Falls alone, without preclinical AD pathology, were associated with a moderate, though not statistically significant, increase in risk (HR = 6.7, 95% CI: 0.8–53.8; *p *= 0.08 in the age‐adjusted model).

**TABLE 2:** Hazard ratios for progression to mild dementia (CDR 1).

Parameter	Model 1, unadjusted^*^		Model 2, controlling for age^*^	
	HR	95% CI	HR	95% CI
Preclinical AD−Fall− (reference)	—	—	—	—
Preclinical AD−Fall+	5.6	0.7–45.0	6.7	0.8–53.8
Preclinical AD+Fall−	15.1	1.8–125.7	17.4	2.1–146.8
Preclinical AD+Fall+	21.3	2.7–166.9	26.9	3.4–215.4
Age	—	—	1.2	1.1–1.3
Concordance (Harrell's C‐index)	0.76 (SE = 0.04)		0.87 (SE = 0.03)	

Abbreviations: AD, Alzheimer's disease; CDR, Clinical Dementia Rating; CI, confidence interval; HR, hazard ratio; SE, standard error.

* Overall model, *p *≤ 0.001.

In the unadjusted Cox model, Harrell's C‐index was 0.76 (SE = 0.044), indicating moderate discriminatory ability. After adjusting for age, the model's performance improved to a C‐index of 0.87 (SE = 0.032), reflecting strong discrimination.

## 4 DISCUSSION

Our findings suggest that falls are associated with faster progression to mild dementia symptoms among older adults with preclinical AD. The Preclinical AD+Fall+ group showed the most rapid progression to CDR 1 and had the HRs of progressing to CDR 1. These results corroborate prior research that highlights motor impairments as potential early indicators of AD progression,^6^, ^16^ and that severe falls may precede an AD dementia diagnosis.^20^

The Kaplan–Meier curves (Figure 1) and HRs (Table 2) demonstrate that those with falls and amyloid pathology may have an accelerated timeline from unimpaired cognition to mild dementia symptoms. This may be particularly notable among *APOE*4 carriers, who comprised a substantial portion of the Preclinical AD+Fall+ group (67%), and have a known elevated risk of AD‐related cognitive decline.^46^, ^47^ *APOE*4 carriers have also been found to have increased gait variability and risk of falls,^48^, ^49^ but this relationship has been less explored. The potential interaction between *APOE*4 status, fall risk, and AD progression may underscore the need for multifactorial approaches to dementia prevention that consider both motor function and genetic risk factors.

Individuals with preclinical AD who did not experience falls (Preclinical AD+Fall−) also had a significantly increased risk of progression to CDR 1, suggesting that amyloid pathology alone is a critical driver of dementia and supports existing literature.^50^, ^51^, ^52^ However, the addition of fall history appears to substantially heighten this risk, as shown by the increased HRs in the Preclinical AD+Fall+ group compared to the Preclinical AD+Fall− group. Falls and their precipitating mechanisms (e.g., gait and balance impairments)^6^, ^16^, ^53^ may capture important impairments that occur in preclinical AD.^6^, ^53^ It is possible that adding these impairments to risk of dementia progression may enhance existing models for risk of AD progression.

These findings emphasize the importance of monitoring fall risk in older adults, particularly those with preclinical AD. They also support that preclinical AD pathology and related impairments in preclinical AD may contribute to a greater vulnerability to fall‐related injuries and may serve as a clinical marker for impending progression to dementia. A better understanding of the increased fall risk in this period could lead to both earlier identification of preclinical AD and the development of targeted fall prevention interventions for those with preclinical AD. Specific mechanisms underlying increased risk of falls in preclinical AD could also lead to insights that distinguish the trajectory of “healthy aging” (without preclinical AD) to the trajectory of aging with preclinical AD.^18^

While the exact relationship between falls and preclinical AD is unknown, there are significant implications to whether preclinical AD increases fall risk, whether falls contribute to preclinical AD, or a combination of the two. There are implications for early AD diagnosis, as even with the advent of blood biomarkers, it is not currently clinically indicated to test for AD pathology before symptom onset,^24^ While this could change if secondary prevention is successful, in the more immediate future, falls could help identify those at risk for AD dementia before subtle cognitive changes are detected. Importantly, falls can easily be identified and recorded by patients and families and discussed with providers compared to going in for a blood test. Meanwhile, screening for fall risk and implementing fall prevention interventions for those with AD pathology may also be beneficial, as falls negatively impact the functioning and quality of life of individuals and families and place substantial burden on healthcare systems.^7^, ^8^

Future research should focus on identifying the mechanistic links between falls, motor, and sensory dysfunction and amyloid accumulation to better understand how these factors interact in the progression of AD. Fall risk screening and fall prevention strategies are generally inexpensive and have added benefits of improving other areas of daily activity functioning,^9^ particularly compared to biomarker assessments of AD pathology and disease‐modifying therapeutics. Falls and fall risk screening could serve as an important, easily identifiable clinical marker that could prompt referral to further neurological testing when indicated. Further, in the future, fall prevention interventions tailored to individuals with preclinical AD may be a particularly timely and beneficial approach. It should also be explored whether preventing falls could delay the onset of dementia in this at‐risk population. On the other hand, the increasing availability of blood tests for AD^54^, ^55^, ^56^ could inform identification of individuals who may be at risk for falls. More widespread access to these tests could identify individuals who may benefit from a falls assessment and could also enable better understanding, monitoring, and ultimately tailored intervention to prevent falls among this population.

Strengths of this study include methods of fall data collection and longitudinal follow‐up of CDR with limited attrition. Prospective fall ascertainment with fall calendar journal and monthly follow‐up affords greater detail and accuracy of fall data that may be prone to less bias compared to self‐report methods often with a long recall period.^29^, ^31^, ^32^, ^33^ Longitudinal follow‐up of CDR affords insight into real‐world dementia symptom progression rather than risk that cross‐sectional or shorter‐term studies can offer.

Limitations of this study include lack of generalizability of the cohort, lack of longitudinal fall data (beyond the first 12 months of the study), small sample sizes due to group stratification reducing statistical power, and varying attrition rates across groups (death 19%–28%; study withdrawal 5%–26%). Falls were not adjusted for other known risk factors beyond age; future studies should include comprehensive covariates. Future work should also include more diverse and representative samples with greater sample sizes, rigorous longitudinal fall data collection, and follow‐up on physical and cognitive status after CDR progression or dementia diagnosis to capture a longer trajectory of functioning through the course of aging with AD. While it is a goal of the Knight ADRC to recruit and retain diverse participants, and efforts are ongoing toward this goal, the participant data used in this study reflects a cohort lacking in diversity.

In sum, these findings suggest falls associate with faster progression of AD dementia, warranting further research to disentangle motor function and falls in people aging without preclinical AD and those with preclinical AD. Falls may be an important marker of the preclinical AD trajectory and signal a more rapid progression to dementia symptoms. Individuals with preclinical AD may also be at a greater risk of falling, and we should consider fall risk screening and tailored fall prevention interventions for these individuals.

## CONFLICT OF INTEREST STATEMENT

T.L.S.B. has received grants or contracts from Siemens paid to her institution; consulting fees from Biogen, Eli Lilly, Eisai, Bristol Myers Squibb, J&J, Merck and Roche; payment for CME activity from Medscape, PeerView, and Neurology Today and payment for webinar activity from Applied Radiology; and travel reimbursement from Cedars Sinai Medical Center, Hong Kong Neurological Association, Alzheimer's Association, American College of Radiology, Radiological Society of North America, Stanford University, J&J, and Eisai. T.L.S.B. reports the following patents planned, issued or pending: US patent 16/097, 457 (DIFFUSION BASIS SPECTRUM IMAGING (DBSI), A NOVEL DIFFUSION MRI METHOD USED TO QUANTIFY NEUROINFLAMMATION AND PREDICT ALZHEIMER'S DISEASE (AD) PROGRESSION), and US Patent 12,016,701 (Quantitative Differentiation of Tumor Heterogeneity Using Diffusion MR Imaging Data). T.L.S.B. has participated on a data safety monitoring board or advisory board of Siemens and served as an external advisor for NIH‐funded studies (no payments). T.L.S.B. has served as the co‐chair of ASNR Alzheimer's, ARIA and Dementia Study Group, and RSNA Quantitative Imaging Committee (QuIC) (all unpaid). T.L.S.B. has served as a committee member of the American College of Radiology/ALZ NET imaging, NIH CNN Study Section Chair, had a leadership or fiduciary role in the ACR Commission on Neurology, and has sered on the FNIH Biomarker Executive Committee (all unpaid). T.L.S.B. has received technology transfer and precursors for radiopharmaceuticals from Avid Radiopharmaceuticals/Eli Lilly, LMI, and Lantheus, as well as a scanner loan from Hyperfine to her institution.  For additional references regarding T.L.S.B. disclosures, see Sunshine ACT reporting here: https://openpaymentsdata.cms.gov/physician/850680

B.M.A. has received technology transfer and precursors for radiopharmaceuticals from Avid Radiopharmaceuticals, Cerveau, and LMI.

J.C.M has received consulting fees from Barcelona Brain Research Center; speaking at AAIM meeting Longer Life Foundation, International Brain Health Symposium, and CBR International Advisory Meeting; travel support from AAIM meeting, Longer Life Foundation, AD/PD meetings, ATRI/ADNI Investigators meeting, ADRC Spring and Fall meetings, DIAN symposium, ADC meeting, International conference on Health Aging & Biomarkers, and AAN Spring; and has served on Cure Alzheimer's Fund, Research Strategy Council and LEADS Advisory Board, Indiana University. All other authors report no conflicts of interest. Author disclosures are available in the supporting information.

## CONSENT STATEMENT

All participants provided written informed consent.

## Supporting information



Supporting Information

Supporting Information
