# Supplementary material for: Falls predict faster progression to Alzheimer's dementia
Source: Alzheimers Dement. 2026 Feb 7;22(2):e71177. doi: 10.1002/alz.71177 (PMC12882545; doi:10.1002/alz.71177)
Supplement: Supplementary file 1 — Supporting Information [file ALZ-22-e71177-s002.docx]

Supplementary Material – Sensitivity Analyses

1. Poisson regression

In the Poisson regression model, the rate of progression to CDR 1 was significantly higher for Preclinical AD+ groups compared to the reference group (Preclinical AD-Fall-). Specifically, Preclinical AD+Fall+ had an IRR of 18.6 (*P*=0.005), Preclinical AD+Fall- had an IRR of 14.4 (*P*=0.014), and Preclinical AD-Fall+ showed a non-significant increase (IRR=5.6, *P*=0.106).

These results are similar to the Cox regression hazard ratios reported in the manuscript with the same reference group, Preclinical AD-Fall-: Preclinical AD+Fall+ had an HR of 21.3 (*P*<.001), Preclinical AD+Fall- had an HR of 15.1 (*P*=.01), and Preclinical AD-Fall+ showed a non-significant increase (HR=5.6, *P*=0.08).

1. Stratified Cox models

We performed stratified Cox models within amyloid-positive participants (Preclinical AD+) and within amyloid-negative participants (Preclinical AD-). These are not adjusted for multiple comparisons.

Among amyloid-negative participants, having a fall was associated with a 2.8-fold higher hazard of conversion compared to no falls, but this was not statistically significant (HR=2.78, 95% CI: 0.34–22.6, *P*=0.34; C-index=0.60). Among amyloid-positive participants, falls were associated with a 2.7-fold higher hazard of conversion, which was also not statistically significant but may signal a trend (HR=2.66, 95% CI: 0.94–7.48, *P*=0.064; C-index=0.67). While neither association reached significance, the effect of falls appeared more consistent in the amyloid-positive group.
